# Computational Model of Effective Thermal Conductivity of Green Insulating Fibrous Media

**DOI:** 10.3390/ma17010252

**Published:** 2024-01-03

**Authors:** Hamidou Sankara, Dominique Baillis, Ousmane Coulibaly, Rémi Coquard, Naïm Naouar, Zahia Saghrouni

**Affiliations:** 1 LaMCoS, INSA-Lyon, CNRS UMR 5259, Université de Lyon, 69621 Villeurbanne, France; dominique.baillis@insa-lyon.fr (D.B.); naim.naouar@insa-lyon.fr (N.N.); 2 Laboratoire de Physique et de Chimie de l’Environnement (ED-ST/LPCE), Université Joseph KI-ZERBO, Ouagadougou 03 BP 7021, Burkina Faso; coulous2005@yahoo.fr; 3 EC2 Modélisation Campus Lyon Tech, 69603 Villeurbanne, France; remi.coquard@ec2-modelisation.fr; 4 Laboratory of Thermal and Energetic Systems Studies (LESTE), National Engineering School of Monastir, University of Monastir, 5019 Monastir, Tunisia; zahiasagrouni@yahoo.fr

**Keywords:** effective thermal conductivity, thermal insulation, numerical model, homogenization, fibrous media

## Abstract

Modelling effective thermal properties is crucial for optimizing the thermal performance of materials such as new green insulating fibrous media. In this study, a numerical model is proposed to calculate the effective thermal conductivity of these materials. The fibers are considered to be non-overlapping and randomly oriented in space. The numerical model is based on the finite element method. Particular attention is paid to the accuracy of the results and the influence of the choice of the representative elementary volume (REV) for calculation (cubic or rectangular parallelepiped slice). The calculated effective thermal conductivity of fibrous media under different boundary conditions is also investigated. A set of usual mixed boundary conditions is considered, alongside the uniform temperature gradient conditions. The REV rectangular slice and uniform temperature gradient boundary conditions provide a more accurate estimate of the effective thermal conductivity and are therefore recommended for use in place of the usual cubic representative elementary volume and the usual mixed boundary conditions. This robust model represents a principal novelty of the work. The results are compared with experimental and analytical data previously obtained in the literature for juncus maritimus fibrous media, for different fiber volume fractions, with small relative deviations of 7%. Analytical laws are generally based on simplified assumptions such as infinitely long fibers, and may neglect heat transfer between different phases. Both short and long fiber cases are considered in numerical calculations.

## 1. Introduction

During the last decade, bio-sourced thermal insulating materials have experienced a strong development, especially concerning building insulating applications. Examples include works on cellulose-based aerogel [1,2] and composite materials using granular cork [3,4] or natural fibers such as hemp shives [5,6,7,8], flax and straw rape [8], palm date fibers [9,10,11,12], and juncus maritimus fibers [13]. One of the most important factors that make composites increasingly attractive is the ability to manage properties by constitutional design [14]. In order to optimize by the design thermal performances of materials, knowledge and modeling of the thermal properties is of primary importance. Most previous research in the literature has focused on the characterization of the thermal properties of these materials, via analytical and experimental methods. The application of the current paper is particularly concerned with green fibrous media. Concerning this material, the recent work of Saghrouni et al. [13] showed that the analytical Glicksman law was the most appropriate by comparison with experimental data. Analytical models are often limited because they are based on approximations. The Glicksman’s model is based on a resistor approach and assumes that the fibers are infinitely long and randomly oriented in the surroundings. Therefore, accurate and robust numerical models are required in order fo comparison with simplified analytical models. This is one of the purposes of the current paper. We propose a numerical model to calculate the effective thermal conductivity of fibrous media and to compare it with Glicksman’s law. Numerical models present an advantage because they are more generalist. With the advancement of computer computational capabilities, numerical analyses have been employed for the computation of heat conduction, for example [15,16,17,18,19]. Li et al. [20] determined both the in- and out-of-plane thermal conductivities of composites using the representative volume element (REV) technique with two-unit cells created at varied scales and periodic boundary conditions. However, as heterogeneous media can have prohibitively large representative elementary volume (REV) sizes due to the randomness of the microstructure (non periodic), it is often necessary to estimate the true effective thermal conductivity (ETC) based on the apparent thermal conductivity (ATC) of computational domains (elementary volume) smaller than RVE, for which different boundary conditions may provide different results [21]. Thus, in this current paper, particular attention is placed on the precision of the numerical results and the influence of the choice and size of the computational representative elementary volume (REV). We propose considering two types of elementary volume: usual cubic or slice. Until now, elementary cubic volumes [21] have generally been considered, and there has been virtually no previous work on the influence of REV volume shape. This is an innovative aspect of this article, which aims to show the influence and importance of the choice of volume shape. The calculated effective thermal conductivity of fibers media under different boundary conditions is also investigated. A set of usual mixed boundary conditions is considered within the computational homogenization framework, alongside the uniform temperature gradient conditions. In the literature, temperatures are usually imposed on opposite sides, as in the hot-guarded plate experiment [22]. Recently, another type of boundary condition was proposed, that of gradients (UTGC) [21], but applied to foams. These boundary conditions have not been applied to fibrous materials. We propose studying these two boundary conditions in this article, which is also an innovative point in this paper. The numerical model is based on the finite element method. The fibers were studied in a vacuum, air, and finally in a mortar. The cases of short and long fibers are considered. Numerical results are compared with experimental and Glicksman analytical laws results previously used in literature for juncus maritimus fibers [13]. Deviations in numerical results are quantified for different fiber volume fractions.

## 2. Theoretical Models for Effective Thermal Conductivity

Heat conduction through heterogeneous media depends on the structure of the materials and the thermal conductivity of each phase. Different methods, including analytical, approximate, and numerical approached, allow for an evaluation of the effective thermal conductivity ($k_{eff}$) based on the assumption of the equivalent homogeneous medium.

### 2.1. Analytical Approach

Recent works [13] dealing with the thermal conductivity of insulating juncus maritimus fibrous mortar composites model tend to show that the analytical law proposed by Schuetz and Glicksman [23] is the most appropriate in comparison with the experimental data. The analytical model proposed by Schuetz and Glicksman [23,24] is based on a serial parallel approach applicable to fibers in fluid phase and open-cell foams (Equation (1)). They assumed infinitely long, nearly randomly oriented fibers. They considered the total length of all fibers per unit volume, which were oriented in some direction within an infinitesimal solid angle, to be constant. This is their definition of “random fibers”. Isotherms are assumed to be planes perpendicular to the heat flux. This means planes parallel to the face of elementary volume at imposed temperatures. The Glicksman model assumes that there is no exchange between the fibers and the surrounding medium. Thus, when the surrounding gas is in a vacuum, the Glicksman model can be used as reference. However, there are approximations when the surrounding medium is not in a vacuum (such as in air or mortar). The effective thermal conductivity of the medium is given by the following:

(1) $$k_{eff}={{(1-f}_{v}\;)k}_{fluid}+f_{v}\;\frac{1}{3}k_{fib}$$

where $k_{fluid}$ is the thermal conductivity of the fluid phase, $k_{fib}$ the thermal conductivity of the solid phase, and $f_{v}$ is the volume fraction of fibers.

Bruggeman’s model [25] used Maxwell’s model for cylindrical particles (Equation (2)). He obtained an expression for the effective thermal conductivity in the following form:

(2) $$k_{eff}=\frac{k_{mat}\left[1-\left(1-\frac{k_{fib}\;}{k_{mat}}\right)\frac{2}{3}f_{v}\delta \right]}{\left[1+\left(\delta -1\right)f_{v}\right]}$$

where $k_{mat}$ is the thermal conductivity of the continuous phase, $k_{fib}$ is the thermal conductivity of the dispersed phase and $f_{v}$ is the volume fraction of the dispersed phase, respectively. δ is a parameter that depends on the shape of the particles: spherical, cylindrical, or flat. For cylindrical particles (fibers): $\delta =\frac{5k_{mat}+k_{fib}}{3(k_{fib}+k_{mat})}$.

### 2.2. Numerical Calculation of the Thermal Conductivity of Fibrous Composites by FEM

The basic idea of computational homogenization techniques is to obtain the effective properties of heterogeneous materials by solving the Fourier heat equation in a representative elementary volume (REV) of the composite materials with appropriate boundary conditions. The simplest way to determine the thermal conductivity of fibrous from numerical approaches consists of solving the steady state energy equation (Fourier law) in a representative sample subjected to a thermal gradient. As a reminder, the Fourier’s law (Equation (3)) is written as follows:

(3) $$\vec{\varphi }=\;-\lambda \vec{grad}T$$

*T* represents the temperature and λ the thermal conductivity of the material. In our approach, a temperature difference is imposed between two sides of a parallelepipedal sample. The numerical simulation allows for the computation of the average flux crossing the sample, i.e., both sides with imposed temperatures, in a direction normal to these faces and in stationary regime. The effective conductivity of the heterogeneous material is then obtained using the Fourier relation:

(4) $$k_{eff}=\;-\frac{L}{S}\frac{Q}{\left|\Delta T\right|}$$

where ΔT/L is the applied thermal gradient in which *L* is the thickness of the sample and *Q* refers to the heat flux through the sample over an area *S*, which is normal to ΔT/L. When using (Equation (4)), the following points should be highlighted:

- First, one-dimensional (1D) heat transfer is considered; therefore, the area *S* must be much larger than the thickness *L* of the sample. To circumvent this constraint, it is common practice to apply adiabatic conditions on lateral boundaries where temperatures are not prescribed (i.e., boundaries parallel to the direction of heat flow).
- Secondly, this formula assumes that a linear variation in temperature prevails through the thickness of the sample. To achieve this, the absolute value of ΔT must be chosen to be sufficiently small.
- (Equation (4)) allows for estimating the effective conductivity in the direction of the imposed temperature gradient. Therefore, in order to evaluate the possible anisotropy of the material thermal behavior, the same calculation must be performed in each of the three Cartesian directions.
- Finally, we also consider that the thermal contacts between the matrix material and the fibers are perfect: there is no thermal contact resistance at the fiber/matrix interfaces.

The main unknown in (Equation (4)) is the heat flux *Q*. *Q* is closely related to the conductivity and to the spatial arrangement of the different phases of the porous material, which govern the heat diffusion paths inside the heterogeneous material. *Q* also depends on the dimensions of REV and on the boundary conditions applied. The solution of the heat equation by means of the finite-element method is a powerful tool whose formulation is particularly well adapted to the estimation of this flux *Q*. This numerical method is implemented in numerous common software. In the present work, we used the ABAQUS FEM software.

### 2.3. FEM Numerical Modeling with ABAQUS Software

The ABAQUS finite-element software have a thermal solver, allowing for solving the heat equation (Fourier law Equation (3)) in transient or stationary regimes, notably inside composite materials. The software is also equipped with an integrated interface for model development and visualization (ABAQUS CAE), which allows for generating and meshing physical models with the prescribed boundary conditions. The model generation could be automatized by means of Python scripts. Such automatization scripts were used in the present work. They allowed us to generate parallelepipedal representative elementary volumes (REVs) composed of arrangements of fibers immersed in a continuous matrix. The fibers were modeled as circular cylinders whose length-to-diameter ratio (χ = L${}_{f}$/D${}_{f}$) could be varied. Moreover, the distribution of the fiber orientation was also monitored. Two different kinds of fiber orientation distribution were considered in the present work:

- Random orientation in the space (“random 3D”);
- Random orientation in a plane (“random 2D”)—this plane may be, for example, parallel to some sides of the parallelepipedal REV.

Additionally, the fiber volume fraction $f_{v}$ was also controlled. Finally, the dimensions of the REV (length, width, height of $L_{x}$-$L_{y}$-$L_{z}$, respectively) could be modified.

**Figure 1:**
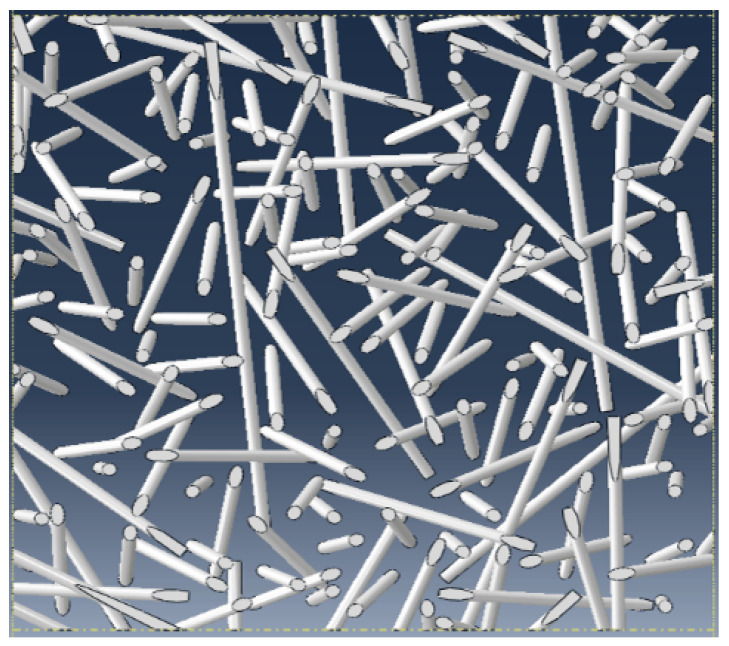
2D visualization of fibers randomly oriented in space.

The algorithm that generated and meshed the REV was based on the following process:

- A first step, conducted before the recourse to the ABAQUS utilization, consisted of generating the arrangement of fibers with the aimed characteristics (fiber volume fraction $f_{v}$, 2D (Figure 1) or 3D random orientations, and diameter distribution DIS($D_{f}$)). The algorithm developed in Fortran language, unfolded as follows:
  1. Random location, orientation, diameter, and length of the new fiber are randomly chosen, i.e., by means of successive random numbers ξ comprised between 0 and 1; 1 < ξ< 0:
    - $x{}_{n}$ = ξ.$L{}_{x}$; $y{}_{n}$ = ξ.$L{}_{y}$; $z{}_{n}$ = ξ.$L{}_{z}$
    - (θ${}_{n}$, φ${}_{n}$, γ${}_{n}$) for a random 3D distribution, we have θ${}_{n}$ = cos${}^{-1}$(ξ); φ${}_{n}$ = 2.π.ξ and γ${}_{n}$ = 0; for a random 2D distribution of fibers in a plane normal to the Z-axis, we have θ${}_{n}$ = π/2; φ${}_{n}$ = 2.π.ξ and γ${}_{n}$ = 0
    - D${}_{n}$ is obtained from a random number ξ in order to satisfy:
      (5) $$\xi =\frac{\int_{0}^{D_{n}}{DIS(D}_{f}).dD_{f}}{\int_{0}{DIS(D}_{f}).dD_{f}}\;$$
    - L${}_{n}$ = χ.D${}_{n}$
  2. Then, we check if this newly generated fiber intersects a previously generated fiber of the current arrangement. In this case, the process returns to Step 1.
  3. Thereafter, the current fiber volume fraction $f_{v,c}$ (fraction of the REV $L_{x}$.$L_{y}$.$L_{z}$ occupied by the fibers) is updated by adding the volume of the new fibers comprised in the REV.

Steps 1–3 are repeated until the volume fraction of the arrangement reaches the desired value ($f_{v,c}\geq \approx f_{v}$).

**Figure 2:**
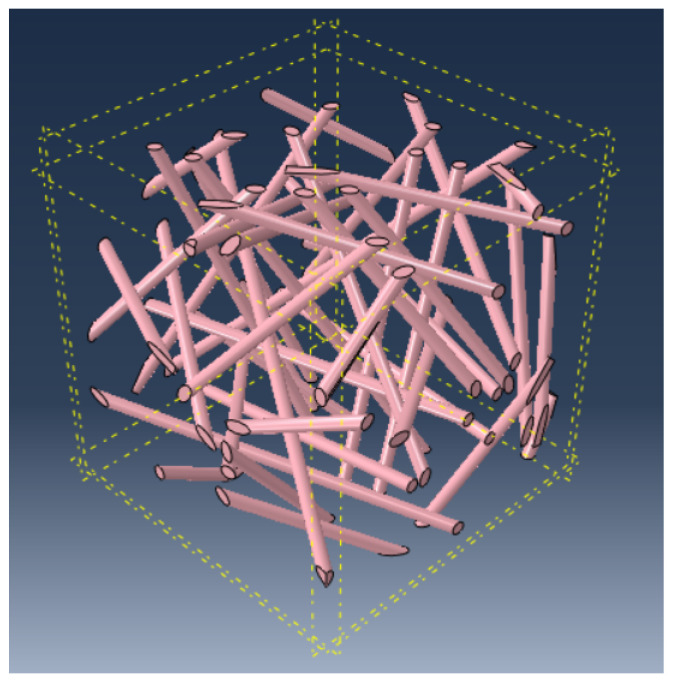
Infinite randomly oriented fibers in a parallelepipedal REV ($L_{x}$ = $L_{y}$ ≈ $L_{z}$).

**Figure 3:**
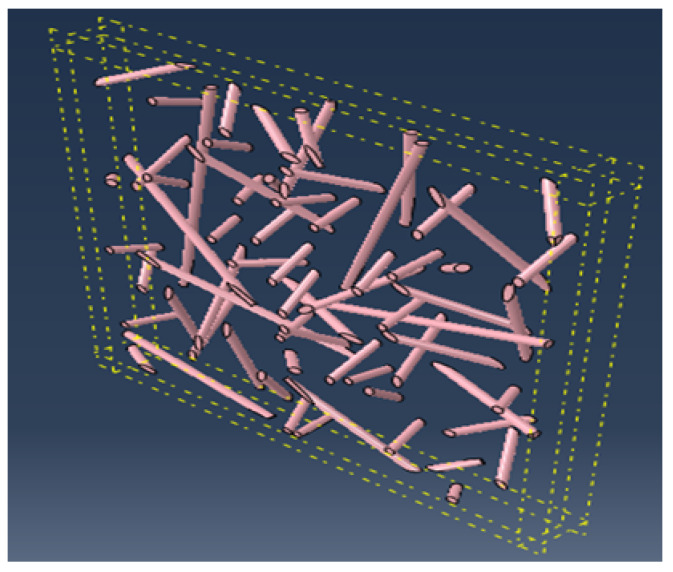
Infinite randomly oriented fibers in a slice REV ($L_{x}$ = $L_{y}$ >> $L_{z}$).

**Figure 4:**
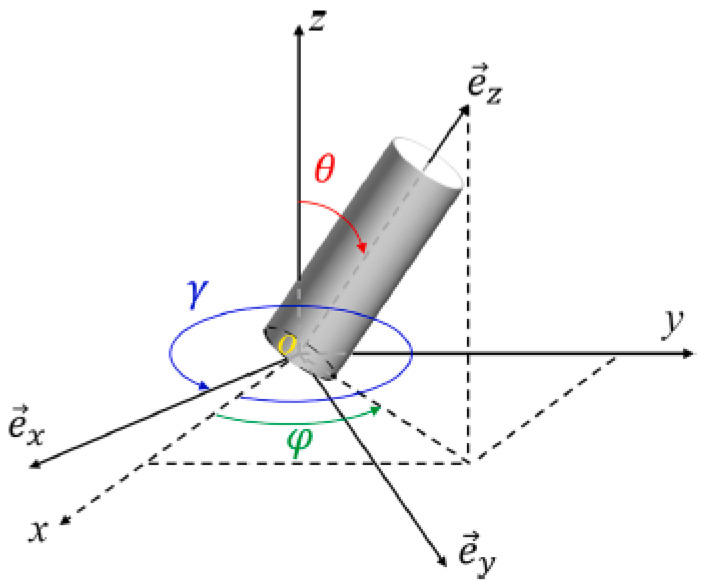
Fiber orientation coordinate system.

Figure 2 and Figure 3 show a cubic REV and a slice REV with randomly oriented fibers, respectively. Figure 4 illustrates the orientation of the fibers in the matrix, where $e_{x}$, $e_{y}$, and $e_{z}$ are the local unit vectors of the Cartesian coordinate system. They can be expressed using the spherical coordinates, as follows:

(6) $$\left\{\begin{array}{c} x=rsin\theta cos\varphi \\ y=rsin\theta sin\varphi \\ z=rcos\theta \end{array}\right.$$

**Figure 5:**
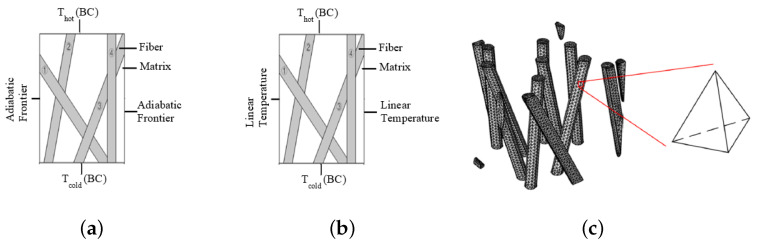
(**a**) Mixed boundary condition (MBC); (**b**) uniform thermal gradient condition (UTGC); (**c**) quadratic mesh.

**Figure 6:**
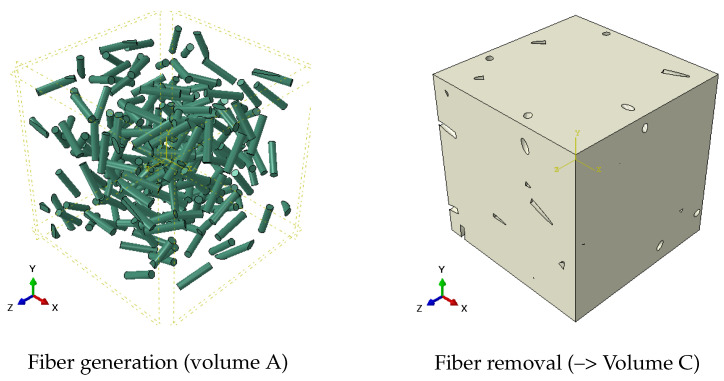

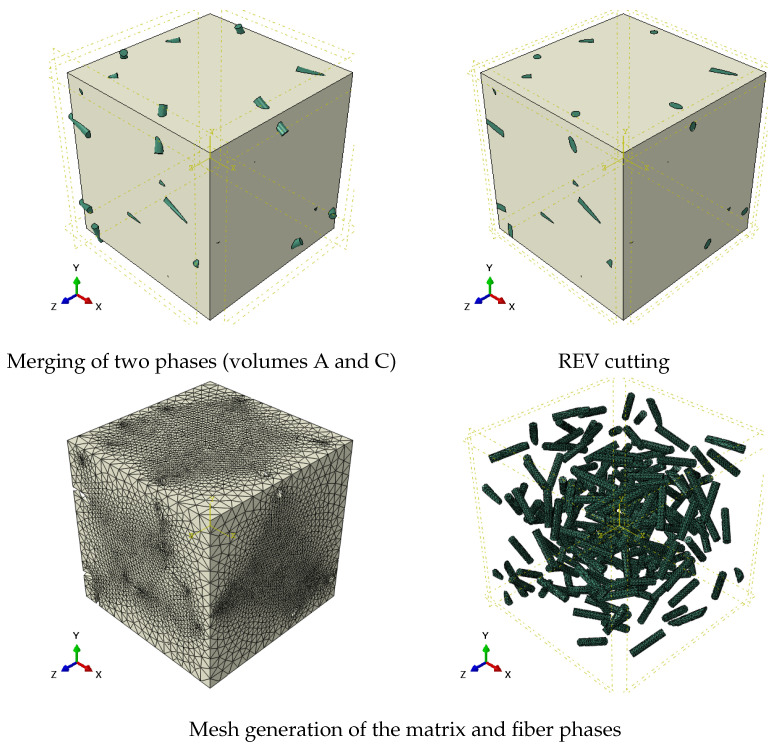
Illustration of the successive steps for the model generation in **ABAQUS CAE**.

- Once the locations and orientations of the fibers in the arrangement with the provided characteristics have been determined, the generation process utilizes the ABAQUS integrated interface (ABAQUS CAE) in order to construct the physical model on which the thermal simulation will be conducted. Thus, several automated steps are successively performed in ABAQUS CAE using Python scripts:
  - Step 1: Geometry generation
    Firstly, a volume containing the N cylindrical objects (each of them with a diameter and length $D_{n}$, $L_{n}$ for *n* = 1, *N*) representing the N fibers of the arrangement is generated. These volumes are created from a homogeneous medium noted A.
    Thereafter, a second parallelepipedal volume (B) with the dimensions of the REV (length–width–height of $L_{x}$-$L_{y}$-$L_{z}$, respectively) containing a homogeneous medium B representing the matrix is generated. A boolean operation (implemented in ABAQUS CAE) is then applied to remove the N cylinders (Volume A) from the parallelepipedal volume (B) to create Volume C. Then, Volumes A and C are gathered together inside a physical model composed of two phases (Volume A: fibers; Volume C: matrix). Finally, still in ABAQUS CAE, this physical model is cut according to the planes x=0; $x=L_{x}$; y=0; $y=L_{y}$; z=0 and $z=L_{z}$ in order to comply with the dimensions of the parallelepipedal REV.
  - Step 2: Meshing of the model
    The meshing tool integrated in the CAE interface is used to generate finite elements of Volumes C (Matrix) and A (N fibers). Quadratic tetrahedron elements («DC3D10» in ABAQUS) illustrated in Figure 5c are used in the present work. They allow for an accurate estimation of the thermal fields with a reasonable mesh refinement. We checked that the mesh densities used were sufficient in order for the results to be independent of the refinement.
  - Step 3: Thermal contact conditions assignment
    As explained previously, the thermal contact at the interface between the matrix and fibers is considered perfect. To account for this hypothesis, in ABAQUS CAE, a thermal continuity condition is specified at the interface between the two volumes (Volume A/Volume C) with a very large thermal conductance leading to a negligible thermal contact resistance. Note that a non-perfect thermal contact between the two phases might be modeled by specifying a lower value of the thermal conductance. The fibers can come into contact with the rest of fibers.
  - Step 4: Material Properties Assignment
    In ABAQUS CAE, the thermal conductivities are affected at each phase ($k_{mat}$ for Volume C representing the Matrix; $k_{fib}$ for Volumes A representing the N fibers) and might depend on the temperature.
    The ABAQUS model is then complete and ready for the realization of the thermal simulations according to prescribed boundary conditions. The different successive steps for the model generation are illustrated in the following Figure 6.

### 2.4. Boundary Conditions Used for Modeling

As indicated previously, the boundary conditions applied to the FEM computations could influence the value of the computed effective conductivity. The two different types of boundary conditions considered in this work are presented in this section. Thus, we use the so-called “mixed” boundary conditions (MBCs) and the “uniform thermal gradient” conditions (UTGC). These two types of boundary conditions have been commonly encountered in previous works on porous materials [26,27,28,29] and sporadically used in computer homogenization [30,31] in order to analyze the macro-homogeneity condition. For MBC (Figure 5a), a hot temperature on one side and a cold temperature on the opposite side are imposed (Equation (8)), while the other four sides are considered adiabatic by the ABAQUS software (Equation (9)):

(7) $$\frac{\partial }{\partial x}\left(k_{x,y,z}\frac{\partial T}{\partial x}\right)+\frac{\partial }{\partial y}\left(k_{x,y,z}\frac{\partial T}{\partial y}\right)+\frac{\partial }{\partial z}\left(k_{x,y,z}\frac{\partial T}{\partial z}\right)=0$$

(8) $${\left.T\right|}_{z=0}=\;T_{cold}\;;\;{\left.T\right|}_{z=Z}=\;T_{hot}$$

(9) $${\left.\frac{\partial T}{\partial x}\right|}_{x=0}=\;{\left.\frac{\partial T}{\partial x}\right|}_{x=X}=\;0\;\;;\;{\left.\frac{\partial T}{\partial y}\right|}_{y=0}=\;{\left.\frac{\partial T}{\partial y}\right|}_{y=Y}=\;0$$

For the UTGC (Figure 5b), the hot temperature on one side and the cold temperature on the opposite side are still prescribed, but on the other lateral sides, we impose a linear temperature variation, which is expressed by the following equations:

(10) $${\left.T\right|}_{z=0}=\;T_{cold}\;;\;{\left.T\right|}_{z=Z}=\;T_{hot}$$

(11) $${\left.T\right|}_{x=0}=\;{\left.T\right|}_{x=X}=\;T_{hot}+\left(\frac{T_{hot}-T_{cold}}{e}\right)z$$

(12) $${\left.T\right|}_{y=0}=\;{\left.T\right|}_{y=Y}=\;T_{hot}+\left(\frac{T_{hot}-T_{cold}}{e}\right)z$$

with Z being the thickness between hot and cold faces.

### 2.5. Estimation of the Effective Conductivity from the FEM Computations

The FEM simulations conducted with both types of boundaryconditions (UTGC or MBC) allow for evaluating the homogeneous effective conductivity of the composite material. Thus, the conduction heat flux density crossing REV can be evaluated numerically from the temperature field in a steady-state regime. In the ABAQUS software, this resulting flux density is directly available for each node at which the temperature is prescribed through the RFL11 variable (so-called «reaction Flux»). The effective thermal conductivity ($k_{eff}$) is then evaluated using (Equation (13)):

(13) $$k_{\mathrm{eff}}=\frac{\left|\;\sum RFL11\right|.e}{S.\Delta T}\;$$

where ∑RFL11 is the total heat flux density crossing the REV from the hot to the cold faces, *S* is the section of the faces, *e* is the thickness of REV according to the temperature gradient, and ΔT is the temperature difference between the two faces. This method was notably successfully used by Coquard et al. [32].

## 3. Results and Discussion

For the present simulations, let us consider a hot temperature $T_{hot}$ = 20 °C on the one side and a cold temperature $T_{cold}$ = 0 °C on the opposite side. The choice of hot and cold temperatures was actually quite arbitrary, and had no influence on the results in terms of thermal conductivity, as the conductivities of the constituent materials (fiber and matrix) used in our calculations were always independent of temperature. As the size of REVs must be large enough to statistically represent the microstructure, and the computation time increases with REV size, it is important to study the influence of REV size to ensure good convergence towards a fair and accurate result, and to determine an appropriate REV size. We considered that the fibers were randomly distributed in the matrix according to a uniform distribution (i.e., there was no preferential orientation). Finally, we approximated the shape of the fibers using cylinders of variable diameter and length, as well as observing a uniform distribution law.

### 3.1. Infinitely Long Fibers in Vacuum

#### 3.1.1. Influence of Mesh Size

In order to select the appropriate mesh size for further simulations, we performed a mesh sensitivity study. We considered a cubic REV with edge 6, a target volume fraction of 4% and 15%, with an infinite fiber length (i.e., Ld = 5000) and a constant fiber diameter of 0.2. We then compared the computed results with the Glicksman relation. The parametric mesh study in a vacuum was conducted considering a solid thermal conductivity with a value of (1 W.K${}^{-1}$.m${}^{-1}$) and a fluid thermal conductivity ${(10}^{-6}$ W.K${}^{-1}$.m${}^{-1}$). In the rest of the article, the value of conductivity ${(10}^{-6}$ W.K${}^{-1}$.m${}^{-1}$) is taken as the vacuum matrix conductivity. So, the finite element mesh construction from our binary image was as follows: (i) a geometric definition of the fluid and solid phases by triangular facets, and (ii) a tetrahedral mesh of the volume. We used four-node linear elements, the ABAQUS DC3D10 heat transfer elements [31]. The meshing step was performed using the open-source code Iso2mesh [33].

**Figure 7:**
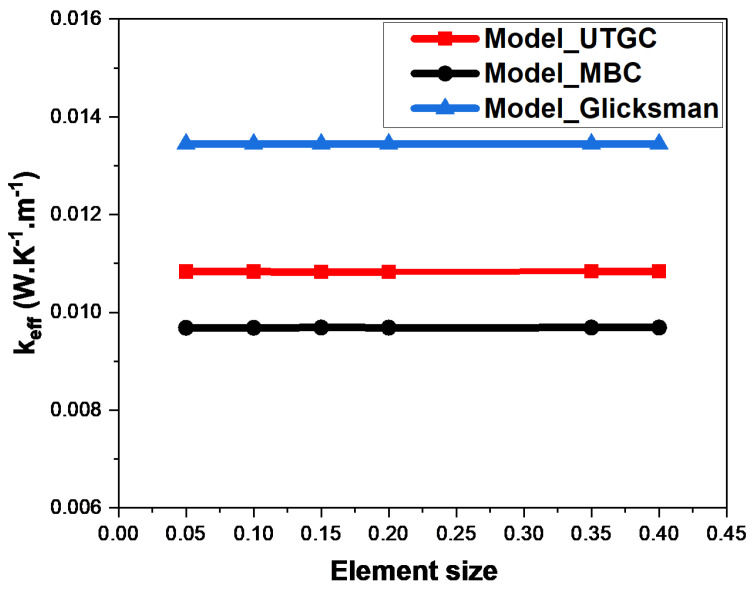
Influence of mesh size for a volume fraction of 4%.

**Figure 8:**
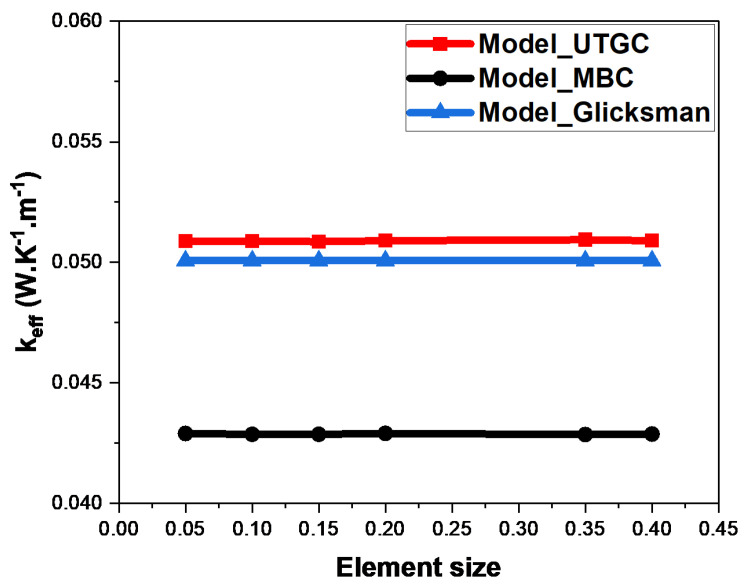
Influence of mesh size for a volume fraction of 15%.

Figure 7 and Figure 8 show the weak influence of the mesh size. For each of the two boundary conditions (MBC or UTGC), when the mesh size increased, the value of thermal conductivity was practically unchanged. The mesh size was sufficiently small to calculate stable and precise results. We chose a mesh size of 0.1 for the rest of our simulations, which constituted a good compromise between the simulation accuracy and calculation time. We observed that the results of the gradient (UTGC) and mixed (MBC) boundary condition computations did not converge to the same value, probably because the REV was not large enough.

#### 3.1.2. Influence of the Cubic REV Size

To obtain more accurate results, we performed calculations in all three directions (X, Y, and Z) through averaging—running the same simulation 10 times to obtain an average value for each parameter.

**Figure 9:**
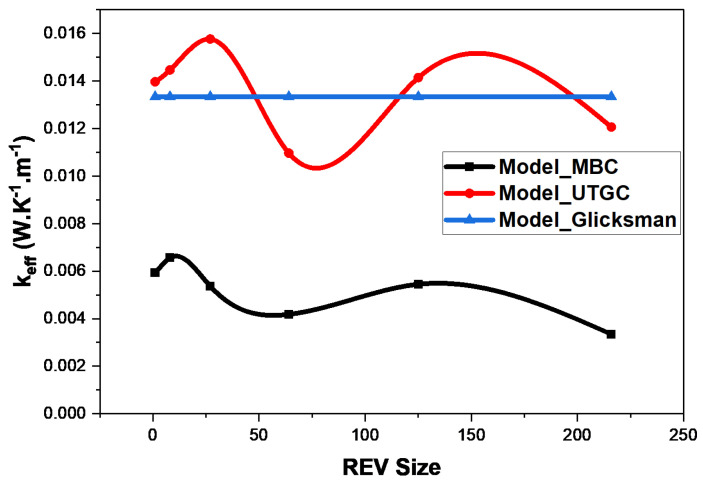
Influence of cubic REV size at 4% volume fraction.

As the fluid is in a vacuum, Glicksman’s thermal conductivity value will tend towards 1/3 of the fibers’ volume fraction. It can be noted that the Glicksman model is appropriate for fibers in a vacuum. Thus, it could be considered as a reference in this case. We can see that the MBC computation tended to converge with a deviation of 75% from the Glicksman model (Figure 9). The volume did not seem to be large enough to have a good convergence, similarly to the conclusions of Z.K Low et al. [21] in the case of foams. UGTC seemed to converge faster but with oscillations towards the Glicksman model as the REV volume increased. These results underline the need to generate larger REVs to converge for more stable and accurate results, as well as the limitations of considering a cubic REV. In the remainder of our study, non-cubic but parallelepiped REVs (small thickness and large surface area, i.e., in slices) will be examined.

#### 3.1.3. Influence of the Slice REV Size

**Figure 10:**
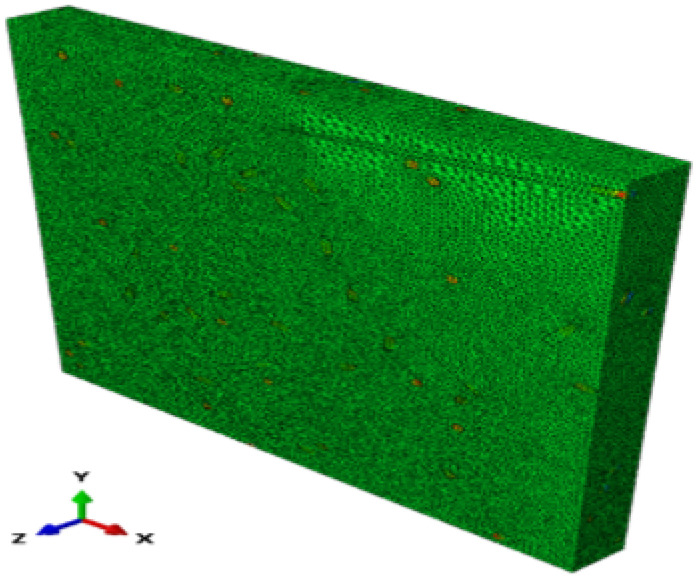
Geometry and meshing of a 3D slice ($L_{x}$ = $L_{y}$ = 10 and $L_{z}$ = 1).

**Figure 11:**
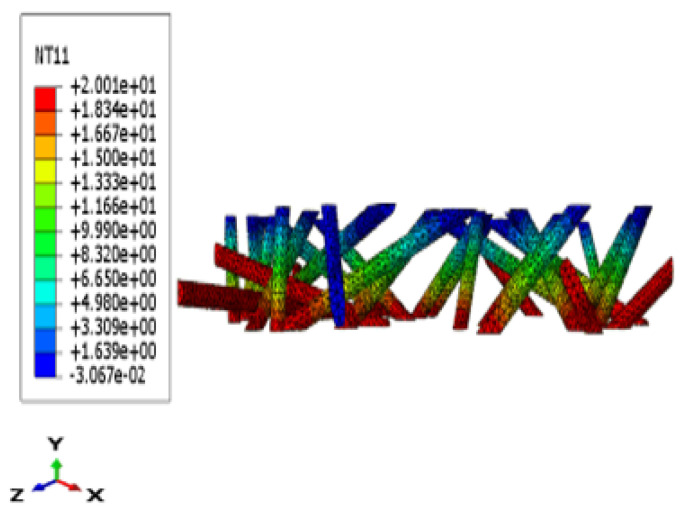
3D fiber temperature fields ($L_{x}$ = $L_{y}$ = 10 and $L_{z}$ = 1).

In this section, we consider a thin slice $L_{z}$ with characteristic surface dimensions ($L_{x}$, $L_{y}$ and $L_{z}$) with $L_{x}\approx L_{y}\;\gg \;L_{z}$. $L_{z}$ is assumed constant, $L_{z}=1$ (Figure 10 and Figure 11).

**Figure 12:**
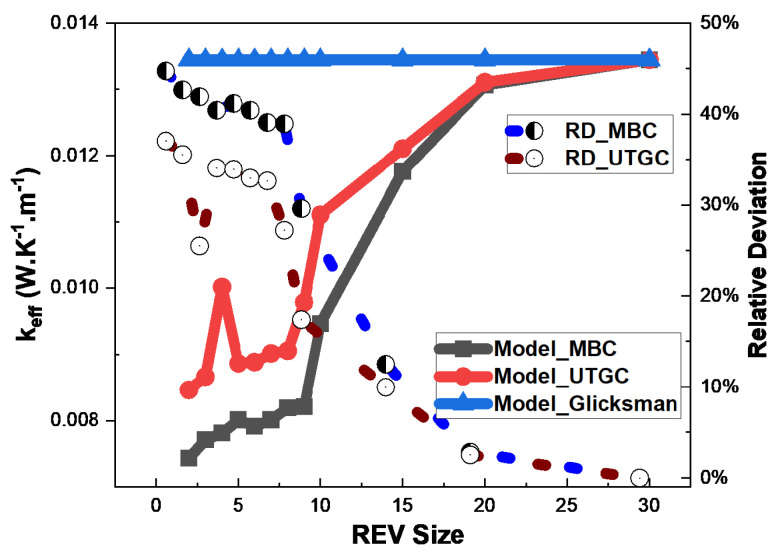
Influence of REV size at 4% volume fraction.

**Figure 13:**
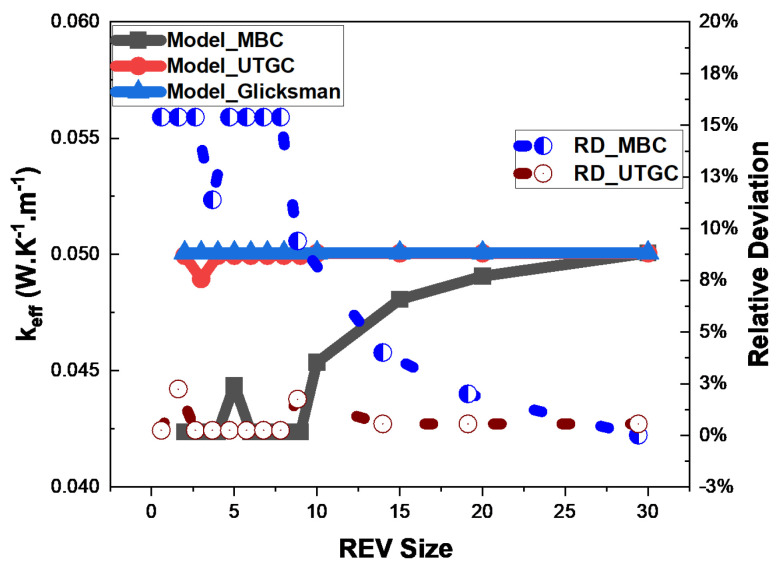
Influence of REV size at 15% volume fraction.

Figure 12 and Figure 13 show the influence of slice REV size ($L_{x}$ = $L_{y}$) for a volume fraction of 4% and 15%. For a fiber volume fraction of 4%, when the lengths $L_{x}\approx L_{y}$ are very close to $L_{z}$, significant fluctuations in effective thermal conductivity ($k_{eff}$) are observed, as was the case for a cubic REV. The curve tends to stabilize when $L_{x}\approx L_{y}$ is greater than 20. For UGTC, for a REV size greater than 20, the deviations are very small, varying between 0% and 5%. UGTC converges faster. Figure 13 shows the influence of the REV size for a fiber volume fraction of 15%, the UGTC model converges better than the MBC model. We can retain that in the case of a slice, the Glicksman model and the numerical model are in very good agreement for $L_{x}\approx L_{y}$ greater than 20. Next, we consider a slice REV with $L_{x}\approx L_{y}=20$.

#### 3.1.4. Influence of Volume Fraction

Fiber volume fraction is an important parameter, as this value can influence the material’s thermal and mechanical properties.

**Figure 14:**
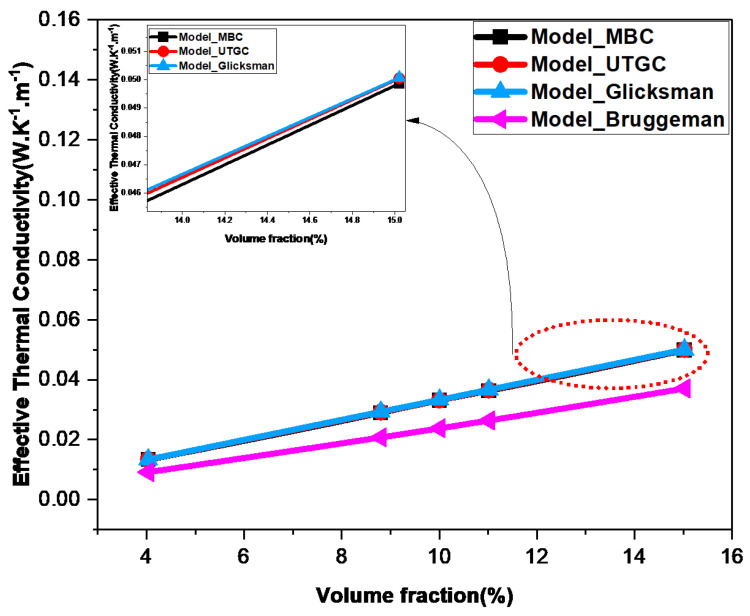
Influence of volume fraction.

Figure 14 shows the variation in ETC as a function of the volume fraction ranging from 4% to 15%. Firstly, the effective thermal conductivity (ETC) of the material increases with the volume fraction. As expected, numerical and analytical results show that thermal conductivity depends linearly on the volume fraction. The effective thermal conductivity (ETC) of the material increases with the volume fraction, because the conductivity of fibers is higher than the air conductivity. It can be observed that there are very small deviations between the values obtained numerically and those calculated with the Glicksman model (deviations of the order of 0.39%). This confirms that the choice of an element size of 0.1 and slice REV with $L_{x}=L_{y}=20$ are appropriate, inducing precise results.

### 3.2. Random Fibers in Air: Infinitely Long and Short Fibers

**Figure 15:**
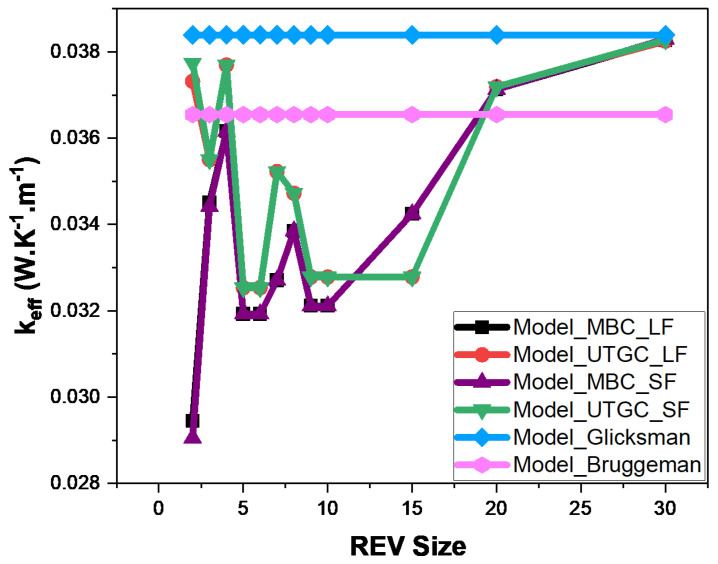
Comparison of numerical and analytical models for increasing the size of REV fibers in air, $k_{fib}$ = 1 W.K${}^{-1}$.m${}^{-1}$ with a volume fraction of 4%.

**Figure 16:**
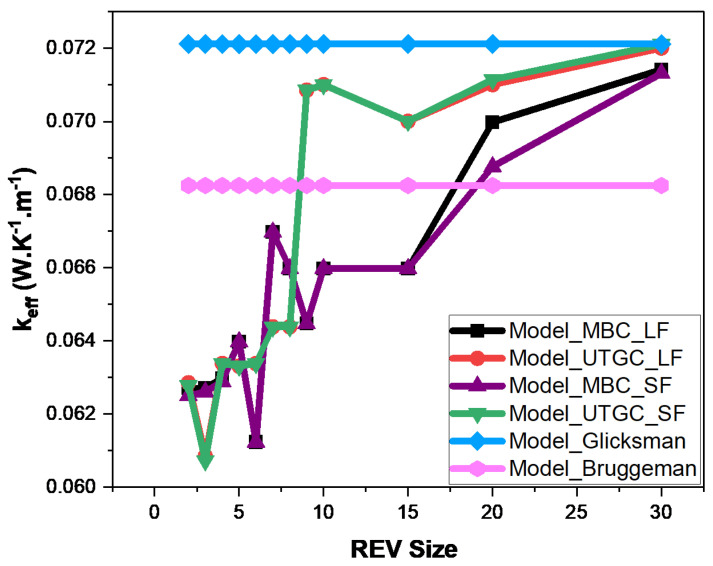
Comparison of numerical and analytical models for increasing the size of REV fibers in air, $k_{fib}$ = 1 W.K${}^{-1}$.m${}^{-1}$ with a volume fraction of 15%.

**Figure 17:**
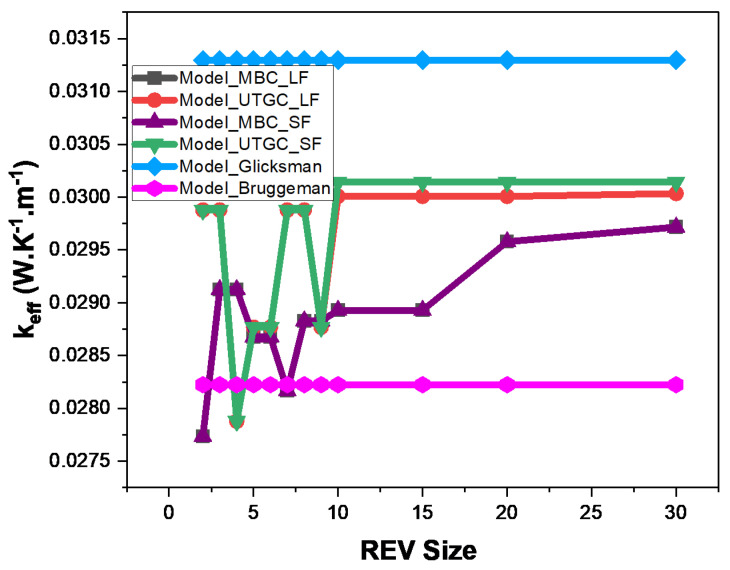
Comparison of numerical and analytical models for increasing the size of REV fibers in air, $k_{fib}$ = 0.472 W.K${}^{-1}$.m${}^{-1}$ with a volume fraction of 4%.

**Figure 18:**
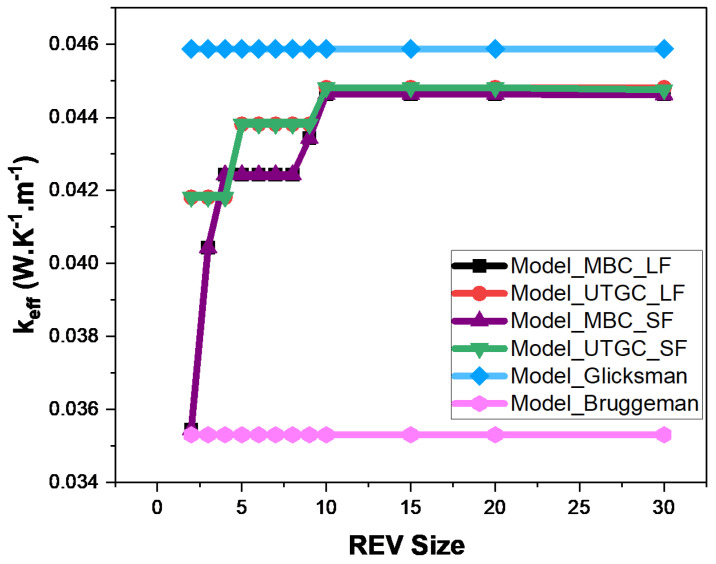
Comparison of numerical and analytical models for increasing the size of REV fibers in air, $k_{fib}$ = 0.472 W.K${}^{-1}$.m${}^{-1}$ with a volume fraction of 15%.

Glicksman’s model is based on the assumption of infinitely long fibers, so it is important to quantify the difference of calculated conductivity between infinitely long fibers and real fibers (not infinitely long). In the case of infinitely long fibers, they extend from one face to another face of parallelepiped REV. In the case of short fibers, there are more embedded fibers in the volume, so there’s more exchange with the surrounding environment. In this section, we considered long and short fibers with lengths of 1000 and 1, respectively, and diameters of 0.2. The fibers were considered in air with volume fractions of 4% and 15%. The conductivity of the air matrix was 0.026 W.K${}^{-1}$.m${}^{-1}$. Simulations were performed with a thermal conductivity of fiber bulk material $k_{fib}$ = 1 W.K${}^{-1}$.m${}^{-1}$ in Figure 15 and Figure 16, as well as considering the thermal conductivity of juntus maritimus fibers ($k_{fib}$ = 0.472 W.K${}^{-1}$.m${}^{-1}$) on Figure 17 and Figure 18. Figure 15, Figure 16, Figure 17 and Figure 18 show the evolution of the effective thermal conductivity as a function of REV size. MBC and UTGC seemed to converge to the same value for increasing REV sizes. The UTGC converged better. In Figure 17 and Figure 18, discrepancies exist with the Glicksman model that remain small, less than 10%. These discrepancies can be explained by exchanges between the air and fibers (especially for $k_{fib}$ = 0.472 W.K${}^{-1}$.m${}^{-1}$) which are not taken into account in the Glicksman model. For the rest of our study, we chose REVs with dimensions of $L_{x}\approx L_{y}=20$ and $L_{z}=1$. We note that the deviations between short (**SF**) and long fibers (**LF**) results remained low.

### 3.3. Fibers in Mortar: Case of Infinitely Long and Short Fibers

This part of the study consists of investigating the numerical model for composite materials and comparing it with previous works on juncus maritimus fibers randomly arranged in the mortar [13]. As the matrix porosity increases with the fiber volume fraction, the thermal conductivity of the matrix needs to be adjusted for each of the fiber volume fractions. For this purpose, we used the experimental values presented in Table 1 for the matrix thermal conductivity [13]. A slice REV of dimension $L_{x}\approx L_{y}=20$ was considered.

**Figure 19:**
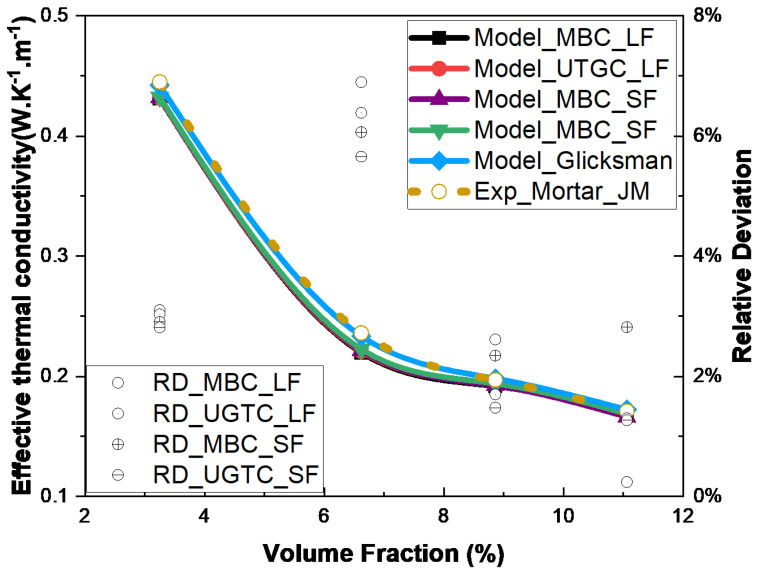
Comparison of the effective thermal conductivities of JM/mortar composites obtained experimentally and numerically for short and long fibers with the Glicksman model calculated using the estimate $k_{JM,fib}$ = 0.472 W.K${}^{-1}$.m${}^{-1}$.

Table 1 shows the thermophysical properties of the various composite samples obtained from the work of Saghrouni et al. [13]. These properties were useful as input data in the numerical simulations. Note that the local thermal conductivity of juncus maritimus was particularly difficult to determine. It was identified in Saghrouni et al. [13] by assuming that Glicksman’s law could be used and by fitting experimental effective conductivities of samples using analytical methods. Thus, it explains the very good agreement between experimental and Glicksman results (Figure 19). Note that Glicksman’s relation was used by replacing the thermal conductivity of the fluid with that of the mortar matrix in Equation (1).

The obtained numerical thermal conductivity results were compared with the experimentally [13] and analytically obtained values in Figure 19. First of all, we can see that the numerical, experimental, and analytical results followed a non-linear decreasing curve. This was due to the thermal conductivity of the mortar varying in a decreasing manner with the increased fiber volume fraction (Table 1). Indeed, the matrix porosity increased with the increased fiber volume fraction. A small discrepancy between the numerical results (MBC, UGTC, and short and long fibers) was observed. The relative differences observed between the numerical models and the Glicksman model were weak, ranging from 0.5% to 7%. These results confirmed that Glicksman’s law can be used as a good approximation, as proposed in [13].

## 4. Conclusions

A new robust numerical approach to calculate the effective conductivity of insulating fibrous media is proposed and compared analytically. Fibers are considered as being randomly oriented. This study provides a useful tool for evaluating the effective thermal conductivity of a desired volume fraction of fibers, and quantifies the deviation from the simplified analytical models. This paper highlights the importance of using two types of boundary conditions, MBC and UGTC, to verify the convergence of the numerical model. It shows that the UGTC boundary conditions model converged faster than the MBC model. The numerical results also show that a slice-type REV was more appropriate for evaluating the effective conductivity than a cubic REV, which can generate errors due to the very large REV size required. The proposed numerical model can be compared with analytical models such as the Glicksman model. The results are compared with experimental and analytical data previously obtained in the literature for juncus maritimus fibrous media for different fiber volume fractions. There are no numerical results on JM’s thermal conductivity in the literature. The analytical model is assumed to be valid, so deviations with an accurate numerical model have to be quantified. The deviations between numerical results for short and long fibers are weak. This paper tends to confirm that the Glicksman analytical law could be used as it has a good approximation with a deviation from numerical results lower than 10%.

## Figures and Tables

**Table 1:** Physical properties of composite samples; JMC1, JMC2, JMC3, JMC4, are the juncus maritimus/mortar samples corresponding to the juncus maritimus mass fractions (2%, 5%, 7%, and 10%), respectively [13].

Sample	JMC1	JMC2	JMC3	JMC4
Mass ratio of juncus maritimus to mortar mixture (%)	2	5	7	10
Juncus maritimus volume fraction (%)	3.25	6.62	8.86	11.06
Mortar thermal conductivity (W.K${}^{-1}$.m${}^{-1}$)	0.454	0.239	0.211	0.182
Composite Mortar/JM Exp (W.K${}^{-1}$.m${}^{-1}$)	0.44	0.236	0.197	0.171
Juncus maritimus Thermal Conductivity (W.K${}^{-1}$.m${}^{-1}$)			0.472	

## Data Availability

The data presented in this study are available on request from the corresponding author.

## Abbreviations

The following abbreviations are used in this manuscript:

ATC	Apparent thermal conductivity
BC	Boundary condition
ETC	Effective thermal conductivity
Exp	Experimental
FEM	Finite element method
JMC	Juncus maritimus composite
LF	Long fiber
MBC	Mixed boundary conditions
RD	Relative deviation
REV	Representative element volume
SF	Short fiber
UTGC	Uniform temperature gradient conditions
**Acronyms**	
*e*	Thickness (m)
*k*	Thermal conductivity (W.K${}^{-1}$.m${}^{-1}$)
*Q*	Heat flow (W)
*S*	Surface (m${}^{2}$)
*T*	Temperature (°C)
**Subscripts**	
eff	Effective
fib	Fiber
fluid	Fluid phase
fv	Volume fraction
JM	Juncus maritimus
mat	Matrix
**Greek symbols**	
χ	fiber length to diameter ratio
Δ	Difference
δ	cylindrical particles (fibers)
